# Integrated Transcriptome and Metabolome Analyses Reveal Candidate Genes Associate with Phenolic Compound Biosynthesis in Different Varieties of *Perilla frutescens*

**DOI:** 10.3390/ijms26072841

**Published:** 2025-03-21

**Authors:** Hong Ye, Jiaxin Mu, Tong Yang, Qi Shen, Yukun Wang

**Affiliations:** 1 Guangdong Provincial Key Laboratory of Utilization and Conservation of Food and Medicinal Resources in Northern Region, Shaoguan University, Shaoguan 512005, China; 19881212hong@163.com; 2 College of Biology and Agriculture, Shaoguan University, Shaoguan 512005, China; muuixyy@163.com (J.M.); sguanxueyuan@163.com (T.Y.); 3 Engineering and Technology Research Center of Shaoguan Horticulture in Shaoguan University, Shaoguan 512005, China; 4 Guangdong Provincial Engineering and Technology Research Center of Special Fruit and Vegetables in Northern Region, Engineering and Technology Research Center of Shaoguan Horticulture in Shaoguan University, Shaoguan 512005, China; 5 School of Pharmaceutical Sciences, Institute of Medical Plant Physiology and Ecology, Guangzhou University of Chinese Medicine, Guangzhou 510006, China

**Keywords:** *Perilla frutescens*, transcriptome, metabolome, phenolic compounds, biosynthesis

## Abstract

*Perilla frutescens* (Perilla) has great potential for utilization in food and medicine due to the abundance of secondary metabolites, especially phenolic compounds. However, the molecular mechanism underlying phenolic compound synthesis in perilla remains poorly understood. By targeted metabolome analysis, we found nine differentially accumulated metabolites among QS2, QS6, and QO7 leaves and nine unique metabolites which only exist in QS6. Furthermore, transcriptome analysis showed the differential expression genes (DEGs) were significantly enriched into phenylpropanoid- and flavonoid-related pathways. Moreover, the integrated transcriptome and metabolome analyses indicated 14 candidates highly correlated with phenolic compound biosynthesis. In addition, phenylpropanoid- and flavonoid-biosynthesis-related DEGs, including one PAL, one CYP73A, one 4CL, two CHI, two F3H, one FLS, three CHS, two CYP75B1, one ANS, and two DFR, were isolated. The results in this study provide useful information for the metabolic regulation of phenolic compounds and serve as essential gene resources for future breeding programs.

## 1. Introduction

Perilla is an annual herb belonging to the *Labiaceae* family and is broadly planted in East Asian regions, especially in China, Japan, Korea, and so on [1]. In China, perilla has been cultivated for more than 2000 years and is known as an important medicinal and edible plant [2]. Leaves of perilla can be eaten with vegetables. As a kind of food, perilla leaves can be cooked as a side dish, boiled into soup, or used as a seasoning for cooking meat to add flavor [2]. As a kind of traditional medicine, perilla has several properties such as antibacterial, antitumor, antioxidative, anti-inflammatory, and antitussive abilities [3,4,5,6,7]. These effects can be mainly attributed to the abundant primary and secondary components in perilla tissues. Specifically, phenolic acids, flavonoids, and volatile oils serve as effective constituents in perilla leaves, stems, and seeds [8,9,10]. Due to these, perilla is utilized as a crucial source of natural pigments, preservatives, and sweeteners [11].

In plants, phenolic compounds are regarded as a complex class of secondary metabolites. At present, over 8000 phenolic compounds have been found [12]. Phenolic compounds can be categorized as simple phenolics, polyphenols, and other phenolic compounds. Simple phenolics include phenolic acids (hydroxybenzoic and hydroxycinnamic acids) and coumarins. Polyphenols are composed of flavonoids and tannins, while other phenolic compounds are made up of stilbenes, lignans, and lignins [13]. Simple phenolic acid contains a single aromatic ring and can be further divided into hydroxybenzoic acids and hydroxycinnamic acids [14]. The biosynthesis of simple phenolic acids derived from cinnamic acid occurs with the participation of shikimate and phenylpropanoid pathways. Based on related studies, simple phenolic acids are found in almost all foods [12]. For instance, simple phenolic acids can be found and consumed in fruits, legumes, oats, cereals, oils, and their byproducts [12,15,16]. However, studies of the biological mechanism of phenolic compounds in perilla varieties are still rare.

As a group of polyphenols, flavonoids are an essential class of secondary metabolites that widely existed in plants. They are thought of as plant growth and development regulators and prominently used in food and medicine [17]. It is well known that flavonoids are derived from the phenylpropanoid metabolic pathway and can be separated into 12 substances such as chalcones, stilbenes, aurones, flavanones, flavones, isoflavones, and so on [17,18,19]. In detail, flavonoids are generated from phenylalanine, which is synthesized through the shikimate pathway [20]. In the general phenylpropanoid pathway, phenylalanine is converted to *p*-coumaroyl-CoA by the catalysis of phenylalanine ammonia lyase (PAL), cinnamic acid 4-hydroxylase (C4H), and 4-coumarate: CoA ligase (4CL) [17]. Then, the downstream flavonoids are formed via the catalysis of corresponding enzymes. Some perilla varieties are rich in several kinds of flavonoids and thus are always used as the materials to study the regulatory mechanism of flavonoid biosynthesis.

In this study, we performed a global survey of phenolic acids in three different perilla varieties using targeted metabolic analysis, and the type and number of phenolic compounds were uncovered, respectively. Through comparative analysis, the differential phenolic compounds, including the unique and common components in three perilla varieties, were screened. Furthermore, comparative transcriptome analysis was carried out, and the corresponding results highlighted differences in three different perilla varieties at transcriptional level. Subsequently, the integrated transcriptome and metabolome analysis not only provided candidate genes but also revealed valuable information for the metabolic engineering of phenolic compound biosynthesis in the leaves of perilla.

## 2. Results

### 2.1. Differences in Phenolic Compounds in Three Perilla Varieties

**Figure 1:**
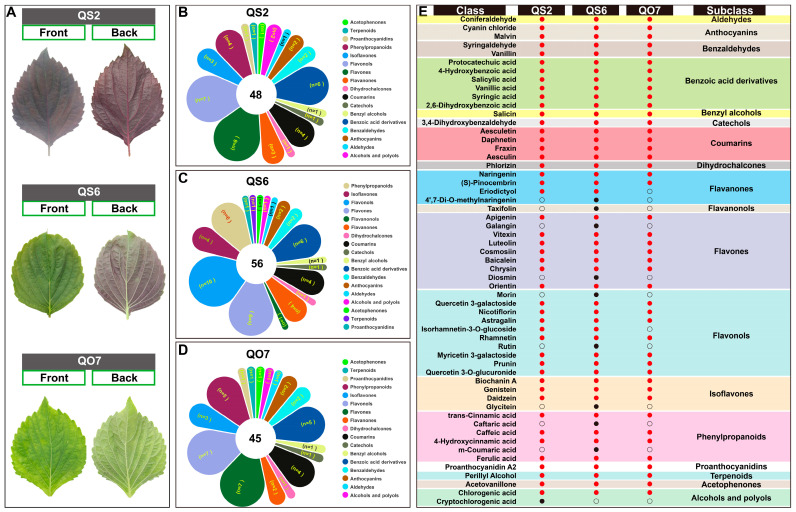
Differences in the species and quantity of phenolic compounds in three perilla varieties. (**A**) Phenotypic characteristics of perilla QS2, QS6, and QO7 leaves. (**B**–**D**) Targeted metabolome analysis of phenolic compounds in three perilla leaves. The total numbers are shown in the center of the figures. (**E**) Details of the species and quantity of phenolic compounds in the three perilla leaves. Red dots indicate phenolic compounds that were found in all the three perilla leaves. Black dots indicate phenolic compounds that were found in only one perilla. Black circles indicate phenolic compounds that were not found in any of the perilla leaves.

Three perilla varieties QS2, QS6, and QO7, which have different leaf colors, were chose as the materials in this study. Both sides of the leaves of QS2 and QO7 were purple and green, respectively, while the front was green and the back was purple in QS6 leaves (Figure 1A), indicating that these varieties have different numbers of pigments, especially anthocyanin. To further understand the reasons for these differences, we performed the targeted metabolome analysis to detect 128 types of phenolic compounds such as anthocyanins, benzoic acid derivatives, flavanones, flavones, isoflavones, phenylpropanoids, terpenoids, and so on. In QS2 leaves, 48 subclasses of metabolites, which belonged to 17 subclasses of phenolic compounds, were detected. Flavones (n = 8), flavonols (n = 7), and benzoic acid derivatives (n = 6) were the top three subclasses of metabolites (Figure 1B). A total of 56 classes of metabolites, which mainly belonged to flavonols (n = 10), flavones (n = 9), benzoic acid derivatives (n = 6), and phenylpropanoids (n = 6), were found in QS6 leaves (Figure 1C). There were also 17 subclasses of phenolic compounds found in QO7 leaves, but the number of metabolite classes (45) was lower than that of QS2 and QS6. Similarly to QS2 and QS6, flavonols (n = 7), flavones (n = 7), benzoic acid derivatives (n = 5), and phenylpropanoids (n = 5) were the main metabolites in QO7 leaves (Figure 1D).

The targeted metabolome analysis can provide absolute quantification data of each metabolite. Next, we compared metabolites in each perilla variety. As shown in Figure 1E, most of the metabolites in QS2, QS6, and QO7 leaves were coincident. Specifically, among 17 subclasses of metabolites, all the perilla leaves contained aldehydes (n = 1), anthocyanins (n = 2), benzaldehydes (n = 2), benzyl alcohols (n = 6), catechol (n = 1), coumarins (n = 4), dihydrochalcones (n = 1), proanthocyanidins (n = 1), terpenoids (n = 1), and acetophenones (n = 1) (Figure 1E). There were clear differences in the components of flavanones (n = 4), flavones (n = 9), flavonols (n = 10), phenylpropanoids (n = 6), isoflavones (n = 4), and alcohols and polyols (n = 2). QO7 leaves had no eriodictyol and isorhamnetin-3-O-glucoside, which were found in QS2 and QS6 leaves. Cryptochlorogenic acid was only detected in QS2 leaves. Moreover, 4′,7-Di-O-methylnaringenin, taxifolin, galangin, diosmin, morin, rutin, glycitein, caftaric acid and m-coumaric acid were only detected in QS6 leaves (Figure 1E). These results suggest that different perilla varieties has different species and quantities of phenolic compounds, especially in flavonoids, and these differences might be an important reason for phenotypic differences between the three kinds of perilla leaves.

### 2.2. Isolation and Enrichment Analysis of Differentially Accumulated Phenolic Compounds

**Figure 2:**
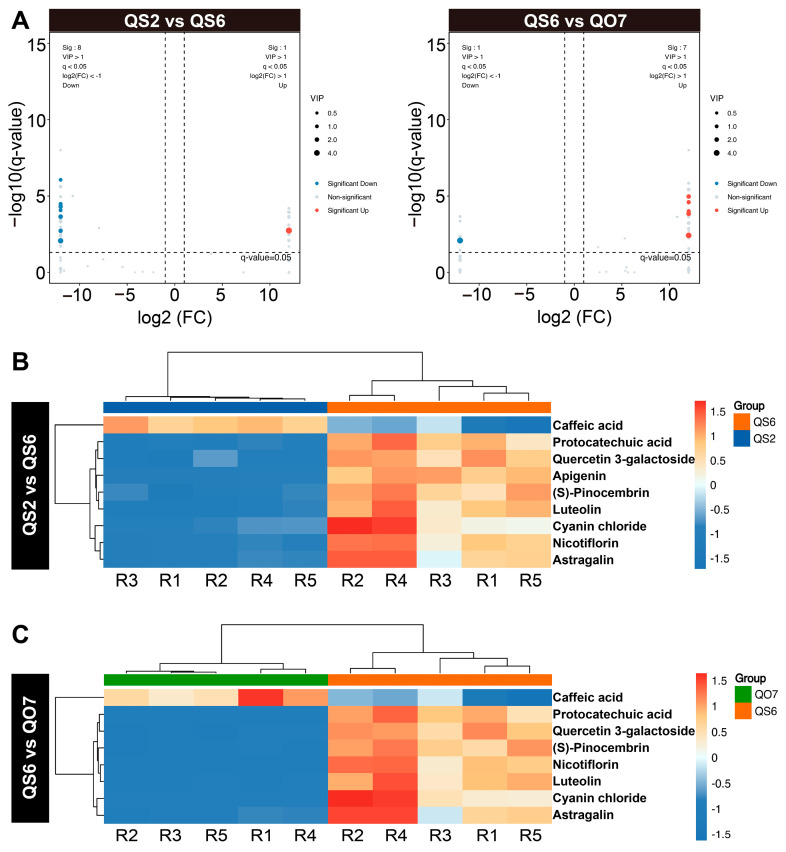
Isolation of differentially accumulated phenolic compounds. (**A**) Volcano plot of differentially accumulated phenolic compounds in comparative groups ‘QS2 vs. QS6’ and ‘QS6 vs. QO7’. (**B**) Heatmap of differentially accumulated phenolic compounds in comparative groups ‘QS2 vs. QS6’. (**C**) Heatmap of differentially accumulated phenolic compounds in comparative groups ‘QS6 vs. QO7’.

**Figure 3:**
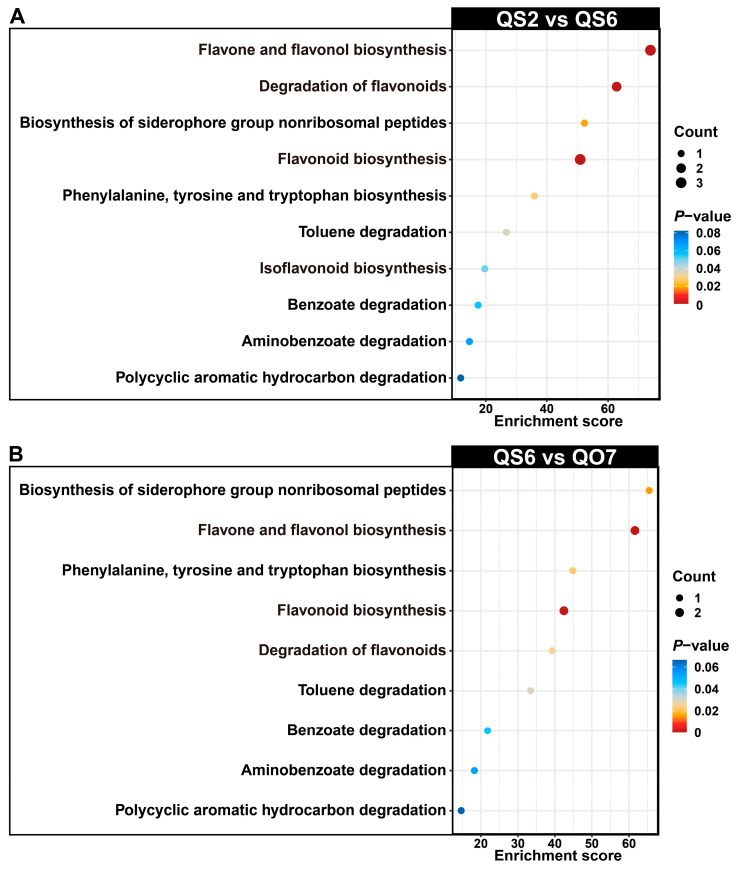
KEGG enrichment analysis of differentially accumulated phenolic compounds in comparative groups (**A**) ‘QS2 vs. QS6’ and (**B**) ‘QS6 vs. QO7’.

Having determined the unique metabolites in QS2 and QS6 (Figure 1E), we then carried out the isolation of differentially accumulated phenolic compounds among the common metabolites. Finally, nine (one up- and eight downregulated) and eight (seven up- and one downregulated) DAMs were screened out from comparable groups ‘QS2 vs. QS6’ and ‘QS6 vs. QO7’, respectively (Figure 2A). The level of caffeic acid in QS6 leaves was the lowest (Figure 2B,C). In addition, levels of protocatechuic acid, quercetin 3-galactoside, apigenin, (*S*)-pinocembrin, luteolin, cyanin chloride, astragalin, and nicotiflorin were the highest in QS6 leaves than in QS2 and QO7 leaves (Figure 2B,C). KEGG enrichment of these DAMs showed that they mainly belonged to ‘flavone and flavonol biosynthesis’, ‘degradation of flavonoids’, and ‘isoflavonoid biosynthesis’ pathways (Figure 3A,B). These results indicated that the activity of the flavonoid biosynthesis pathway in QS2, QS6, and QO7 was inconsonant and the differences in levels of flavonoids might be another reason for phenotypic differences in the three types of perilla leaves.

### 2.3. Comparative Transcriptome Analysis

**Figure 4:**
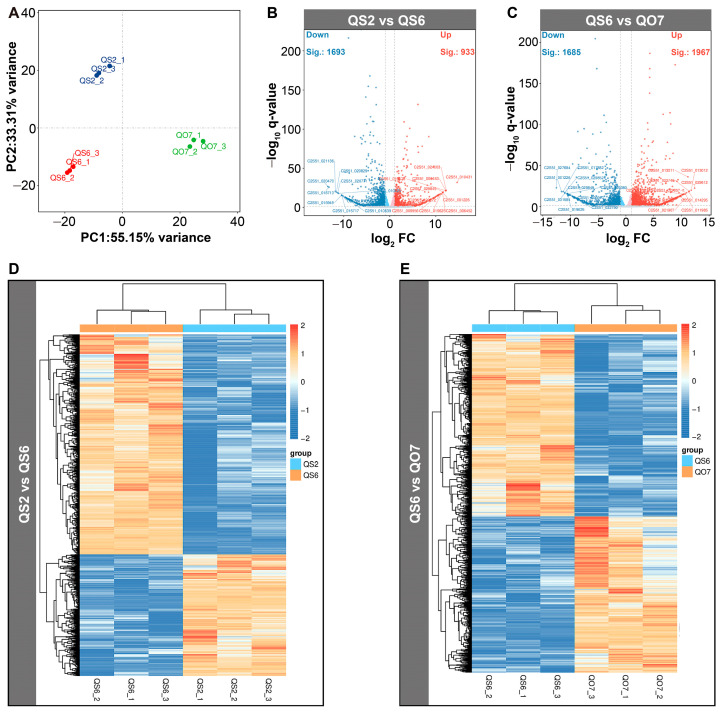
Comparative transcriptome analysis of DEGs. (**A**) PCA of samples used for transcriptome analysis. (**B**) Volcano plot of the DEGs in comparative group ‘QS2 vs. QS6’. (**C**) Volcano plot of the DEGs in comparative group ‘QS6 vs. QO7’. (**D**) Heatmap of the DEGs in comparative group ‘QS2 vs. QS6’. (**E**) Heatmap of the DEGs in comparative group ‘QS6 vs. QO7’.

In order to study more information at the transcriptional level, we performed a comparative transcriptome analysis among QS2, QS6, and QO7 leaves. PCA of gene expression profiles of nine analyzed samples was performed. It showed that the clear distinction between QS6 and QO7 samples was largely accounted for by PC1 (55.15%), while the difference between QS2 and QS6 samples was largely accounted for by PC2 (23.31%) (Figure 4A). In the comparative group ‘QS2 vs. QS6’, 2626 DEGs, including 933 up- and 1693 downregulated genes, were screened out (Figure 4B). In the comparative group ‘QS6 vs. QO7’, 1967 upregulated and 1685 downregulated genes were isolated, respectively (Figure 4C). The expression heatmaps of DEGs in ‘QS2 vs. QS6’ and ‘QS6 vs. QO7’ indicated that each DEG was expressed relatively steadily in each replication and the DEGs in two comparative groups were clustered into two modules displaying clear differences in expression patterns (Figure 4D,E).

**Figure 5:**
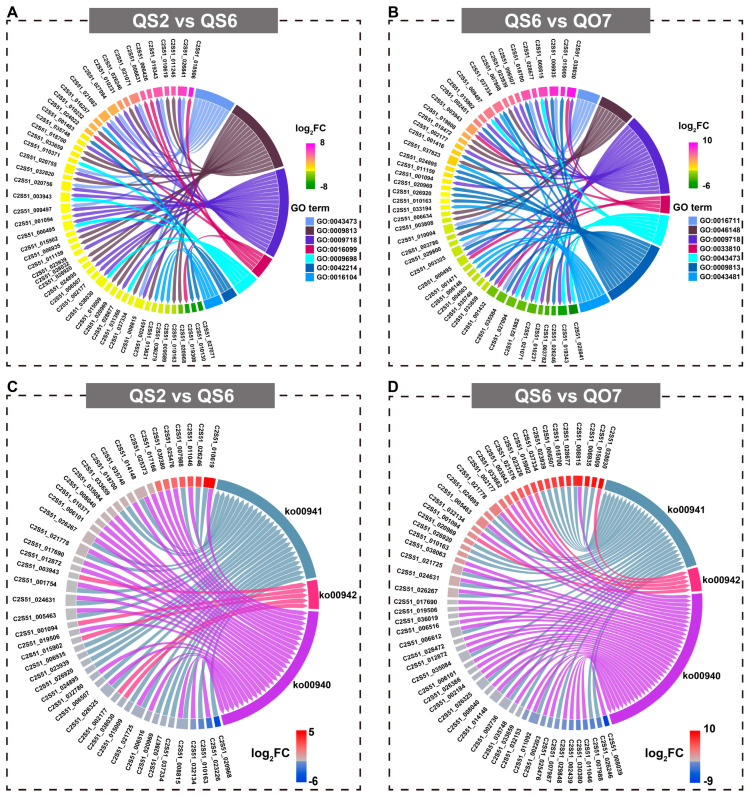
GO and KEGG enrichment analysis of DEGs. (**A**) GO analysis of DEGs in comparative group ‘QS2 vs. QS6’. (**B**) GO analysis of DEGs in comparative group ‘QS6 vs. QO7’. (**C**) KEGG analysis of DEGs in comparative group ‘QS2 vs. QS6’. (**D**) KEGG analysis of DEGs in comparative group ‘QS6 vs. QO7’.

Then, GO enrichment analysis of DEGs was carried out to study the possible functions of DEGs. In the comparative group ‘QS2 vs. QS6’, 52 non-redundant DEGs were involved in pigmentation (GO:0043473; nine genes), flavonoid biosynthetic process (GO:0009813; 17 genes), anthocyanin-containing compound biosynthetic process (GO:0009718; 20 genes), monoterpenoid biosynthetic process (GO:0016099; five genes), phenylpropanoid metabolic process (GO:0009698; six genes), terpene metabolic process (GO:0042214; three genes), and triterpenoid biosynthetic process (GO:0016104; four genes) (Figure 5A). In the comparative group ‘QS6 vs. QO7’, 48 non-redundant DEGs were enriched into flavonoid 3′-monooxygenase activity (GO:0016711; five genes), pigment biosynthetic process (GO:0046148; eight genes), anthocyanin-containing compound biosynthetic process (GO:0009718; 19 genes), anthocyanin 5-O-glucoside 6‴-O-malonyltransferase activity (GO:0033810; four genes), pigmentation (GO:0043473; seven genes), flavonoid biosynthetic process (GO:0009813; 16 genes), and anthocyanin accumulation in tissues in response to UV light (GO:0043481; five genes) (Figure 5B). The results of KEGG pathway analysis demonstrated that several DEGs were clustered into phenylpropanoid biosynthesis (ko00940), flavonoid biosynthesis (ko00941), and anthocyanin biosynthesis (ko00942) pathways. In the comparative group ‘QS2 vs. QS6’, 29, 26, and 5 DEGs were involved in phenylpropanoid biosynthesis, flavonoid biosynthesis, and anthocyanin biosynthesis pathways, respectively (Figure 5C). For the comparative group ‘QS6 vs. QO7’, 37, 27, and 5 DEGs were involved in phenylpropanoid biosynthesis, flavonoid biosynthesis, and anthocyanin biosynthesis pathways, respectively (Figure 5D).

### 2.4. qRT-PCR Validation of DEGs

**Figure 6:**
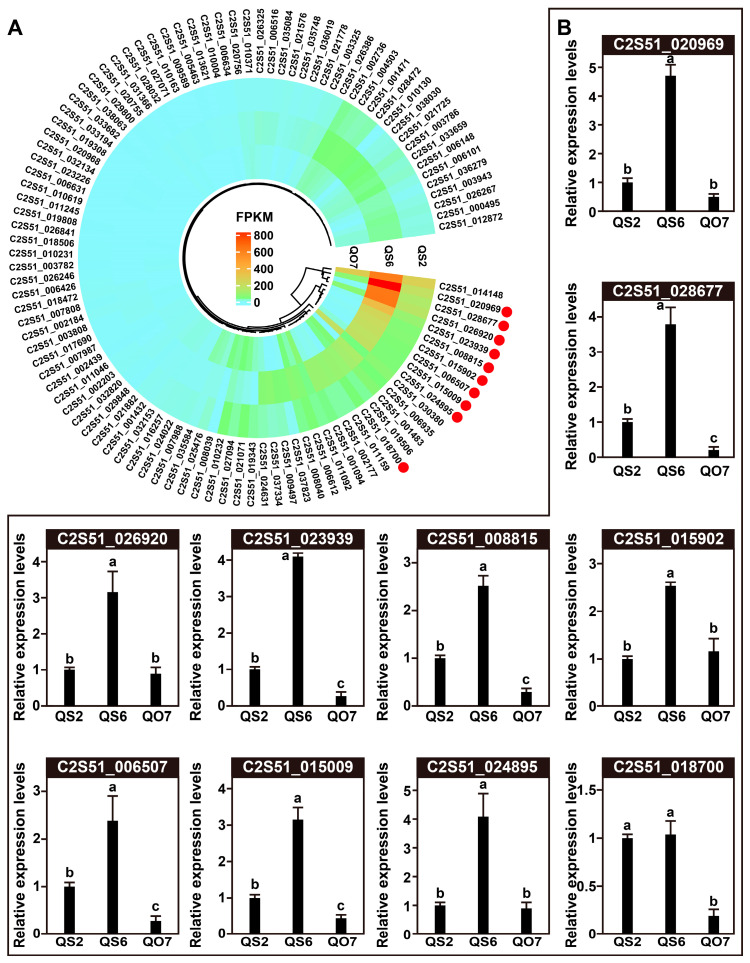
Validation of selected DEGs. (**A**) Expression heatmap of DEGs based on the GO and KEGG enrichment analysis results. Red dots indicate 10 selected DEGs for validation. (**B**) qRT-PCR validation of 10 selected DEGs. Data are presented as means ± standard deviations (n = 3). Lowercase letters are used to indicate differences. Identical letters mean that the differences are not significant, while different letters indicate significant differences.

Based on the results of the GO and KEGG analyses, we combined the DEGs in GO terms and KEGG pathways and finally obtained 104 candidates. We surveyed the expression abundance of each DEG and built a heatmap using the FPKM values. As shown in Figure 6A, 70 candidates were clearly separated into two clades, of which 31 DEGs displayed distinct differences in QS2, QS6, and QO7 leaves, and 39 DEGs showed relatively low FPKM values in QS2, QS6, and QO7 leaves. In order to validate the quality of RNA-seq in this study, we chose 10 DEGs and performed a qRT-PCR assay. The results revealed that all the selected genes showed similar expression patterns in qRT-PCR analysis as that in RNA-seq data (Figure 6B), suggesting that the expression data obtained by RNA-seq of this study were highly reliable.

### 2.5. Integrated Analysis of Transcriptome and Metabolome Data

**Figure 7:**
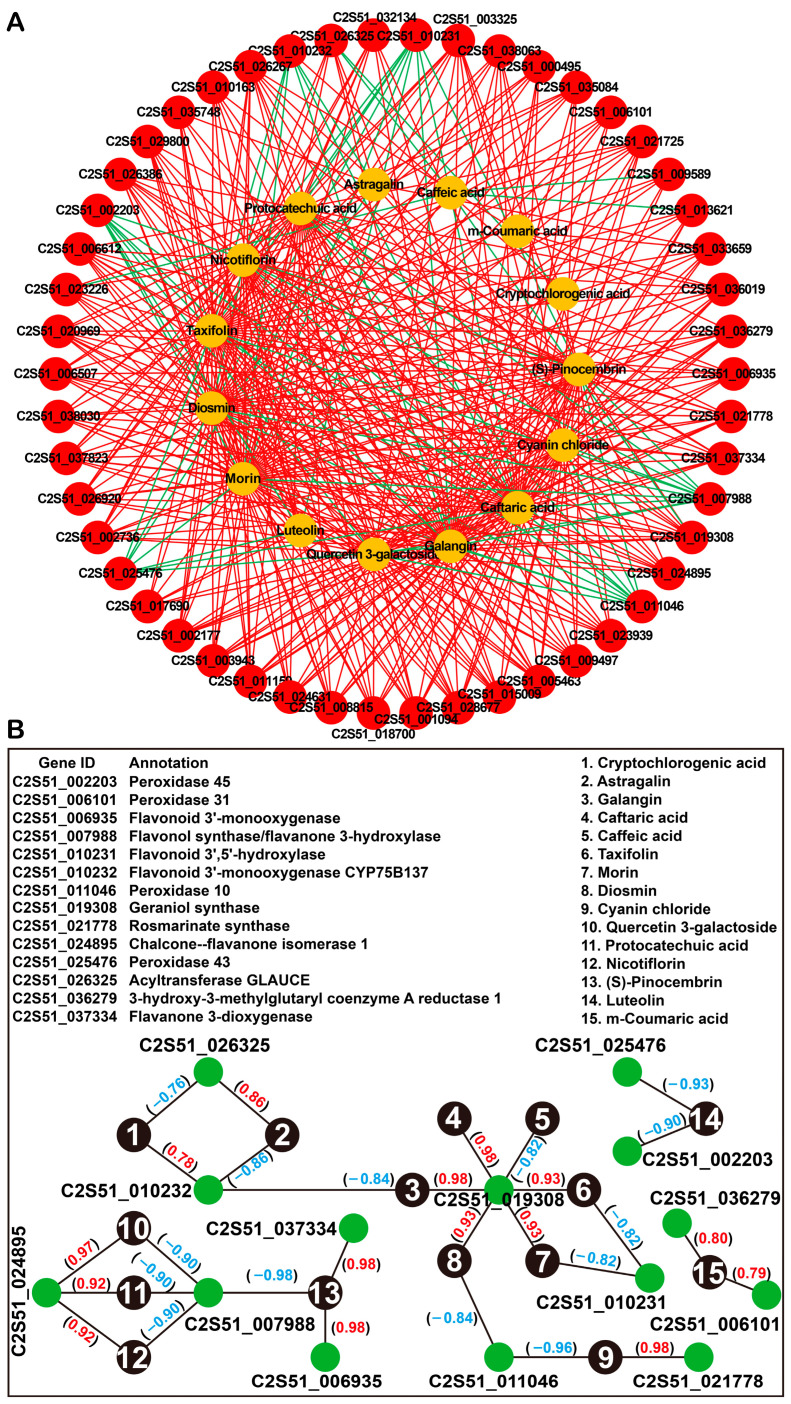
Integrated analysis of the transcriptome and metabolome data. (**A**) Correlation network of DEGs and DAMs. Data showed the top 50 DEGs. (**B**) Correlation network of DEG and DAM pairs that had the highest correlation, including positive (red) and negative (blue) correlations. The annotation of each DEG was shown as well.

To further investigate the relationship between DEGs and DAMs, the coexpression network analysis of the transcriptome and metabolome was conducted. We analyzed the correlations between 50 DEGs (top 50) and 15 phenolic compounds (including 8 DAMs, 6 unique metabolites in QS6, and 1 unique metabolite in QS2). Among these 50 DEGs, 5 DEGs (C2S51_010231, C2S51_002203, C2S51_025476, C2S51_011046, and C2S51_007988) were negatively correlated with the relative phenolic compounds, while 4 DEGs (C2S51_026325, C2S51_010232, C2S51_013621, and C2S51_009589) were positively and negatively correlated with the relative phenolic compounds. The rest of 41 DEGs were all positively correlated with the relative phenolic compounds. In addition, each DEG was correlated with at least six kinds of phenolic metabolites (Figure 7A). These results not only indicated that one DEG might be involved in several phenolic metabolisms, but also suggested that one phenolic metabolite might be regulated by multiple factors.

Based on the Pearson correlation coefficient, we chose the top two DEGs with the highest or lowest Pearson correlation coefficient for each phenolic metabolite. The DEG (C2S51_010232) that encoded a flavonoid 3′-monooxygenase CYP75B protein showed a positive correlation (r = 0.78) with cryptochlorogenic acid, but displayed a negative correlation (r = −0.86) with astragalin. In addition, this gene was also negatively correlated (r = −0.84) with galangin (Figure 7B). C2S51_026325 encoded an acyltransferase GLAUCE and was positively correlated (r = −0.76) with astragalin but negatively correlated (r = 0.86) with cryptochlorogenic acid (Figure 7B). C2S51_019308, which encoded a geraniol synthase, was positively correlative with galangin (r = 0.98), caftaric acid (r = 0.98), taxifolin (r = 0.93), morin (r = 0.93), diosmin (r = 0.93), while negatively correlative with caffeic acid (r = −0.82) (Figure 7B). C2S51_011046 encoded a peroxidase 10 and was negatively correlative with diosmin (r = −0.84) and cyanin chloride (r = −0.96) (Figure 7B). For cyanin chloride, the rosmarinate synthase encoding gene C2S51_021778 showed a positive correlation (r = 0.98) (Figure 7B). The flavonol synthase encoding gene C2S51_007988 was negatively correlative with quercetin 3-galactoside (r = −0.90), protocatechuic acid (r = −0.90), nicotiflorin (r = −0.90), and (S)-Pinocembrin (r = −0.98) (Figure 7B). For (S)-Pinocembrin, a flavonoid 3′-monooxygenase (C2S51_006935) and a flavanone 3-dioxygenase (C2S51_037334) might be the positive regulators (Figure 7B). Chalcone-flavanone isomerase (C2S51_024895) was a positive regulator for quercetin 3-galactoside (r = 0.97), protocatechuic acid (r = 0.92), and nicotiflorin (r = 0.92). Furthermore, two peroxidase-encoding genes C2S51_002203 and C2S51_025476 were negatively correlative with luteolin, and the Pearson correlation coefficients were −0.90 and −0.93, respectively (Figure 7B). Finally, peroxidase-encoding gene C2S51_006101 and 3-hydroxy-3-methylglutaryl CoA reductase encoding gene C2S51_037334 were positively correlative with *m*-coumaric acid, and the Pearson correlation coefficients were 0.79 and 0.80, respectively (Figure 7B). These results provided several potential candidates for phenolic metabolisms in perilla, but their precise functions should be studied in the future.

### 2.6. Profiles of Key Genes and Metabolites Associated with Phenolic Metabolisms in Perilla

**Figure 8:**
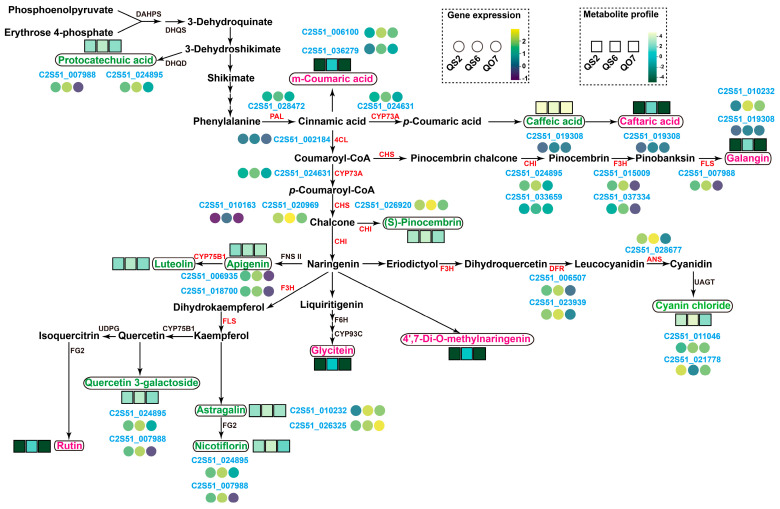
Expression profiles of genes and metabolites involved in shikimate, phenylpropanoid, and flavonoid biosynthesis pathways. The circular patterns represent the expression levels of genes in QS2, QS6, and QO7 leaves. The rectangle patterns with different colors represent the accumulations of metabolites. Metabolites labeled by green color indicate DAMs. Metabolites labeled by pink color represent the unique one only in QS6.

Phenolic compounds are known as the most prevalent secondary metabolites, synthesized in all plant cells [21,22]. In line with this, our abovementioned results also showed that many kinds of phenolic metabolisms, such as flavanols, flavanones, flavanonols, flavones, flavonols, isoflavones, phenylpropanoids, and anthocyanins, were synthesized in perilla leaves (Figure 1, Figure 2 and Figure 3). The biosynthesis of simple phenolic acids derived from cinnamic acid needs the combined action of shikimate and phenylpropanoid pathways. The shikimate pathway is the metabolic way for the synthesis of phenylalanine, tyrosine, and tryptophan [23,24]. For the synthesis of benzoic acids, shikimate and phenylpropanoid pathway intermediates are used as precursors [25]. As a kind of benzoic acid derivative, protocatechuic acid is formed from dehydration and hydroxylation of 3-dehydroshikimate [26]. In this study, we found that the content of DAM protocatechuic acid was the highest in QS6 leaves (Figure 2B,C and Figure 8). However, we found that the expression levels of 3-Dehydroquinate dehydratase (DHQD) encoding gene (C2S51_002722) did not reach a significant level (Figure 8). In addition, the direct regulatory factors of the DAMs caffeic acid, apigenin, cyanin chloride, astragalin, nicotiflorin, and quercetin 3-galactoside were not detected (Figure 8), indicating that the biosynthesis of these metabolites might be regulated by key enzymes in upstream pathways. A similar situation was observed in the biosynthesis of unique metabolites (only detected in QS6), such as *m*-coumaric acid, caftaric acid, rutin, glycitein, and 4′,7-Di-O-methylnaringenin (Figure 8). Given these, it was worth confirming the functions of the DEGs that showed high correlation with metabolites mentioned above (Figure 7 and Figure 8). Moreover, we found that most of the DAMs and unique metabolites (only detected in QS6) were involved in phenylpropanoid biosynthesis and flavonoid biosynthesis pathways (Figure 8). A phenylalanine ammonia lyase (PAL) encoding gene C2S51_028472, which was highly expressed in QS6 leaves, was screened out as the initiator enzyme of phenylpropanoid biosynthesis pathway (Figure 8). PAL catalyzes the deamination of phenylalanine to cinnamic acid; thus, as the derivative of cinnamic acid, *m*-coumaric acid might be controlled by PAL. In addition, caffeic acid and caftaric acid, which were derived from cinnamic acid in phenylpropanoid biosynthesis pathway, might be directly regulated by trans-cinnamate 4-monooxygenase (CYP73A) encoded gene (C2S51_024631) (Figure 8).

By performing integrative analysis of transcriptome and metabolome (Figure 7), we concluded that flavonoid metabolism, especially flavones and flavonols, showed different patterns in QS2, QS6, and QO7 leaves. Therefore, we isolated several essential genes involved in flavonoid biosynthesis pathway (Figure 8). Based on the KEGG pathway database, a brief flavonoid biosynthetic pathway was established in this study (Figure 8). The enzymes involved in the flavonoid pathway were isolated as follows: 4-coumarate-CoA-ligase (4CL, C2S51_002184), chalcone synthase (CHS, C2S51_010163, C2S51_020969, C2S51_026920), chalcone isomerase (CHI, C2S51_024895, C2S51_033659), naringenin 3-dioxygenase (F3H, C2S51_015009, C2S51_037334), flavonol synthase (FLS, C2S51_037334), bifunctional dihydroflavonol 4-reductase/flavanone 4-reductase enzymes (DFR, C2S51_006507, C2S51_023939), anthocyanidin synthase (ANS, C2S51_028677), and flavonoid 3′-monooxygenase (CYP75B1, C2S51_006935, C2S51_018700) (Figure 8). Moreover, we noticed that these flavonoid biosynthesis genes had different expression patterns among the three perilla leaves. The expression levels of these structural genes were the highest in QS6 leaves compared to the QS2 and QO7 leaves (Figure 8).

## 3. Discussion

### 3.1. New Insights into the Constitution of Phenolic Compounds in Perilla Leaves

As a kind of edible and medical herb, the nutritive value and effective constituents of perilla have been studied widely. So far, 271 types of phytochemical compounds have been isolated and reported in perilla tissues [3]. Among these phytochemical compounds, phenolic compounds occupy a certain proportion. By using capillary electrophoresis, Peng et al. reported that catechin, ferulic acid, apigenin, luteolin, rosmarinic acid, and caffeic acid are major important active ingredients in perilla leaves and seeds [10]. In addition, a considerable number of phenolic compounds such as gallic acid, isovanillic acid, chlorogenic acid, sinapic acid, and quercetin have been detected in aqueous and organic solvent extracts of perilla [27,28].

In this study, we chose three perilla varieties QS2, QS6, and QO7, which have phenotypical differences in leaf colors (Figure 1A). We aimed to find differences in phenolic compounds among these perilla varieties by using targeted metabolome analysis, which is an efficient way to identify qualitative and quantitative information of metabolites. The results showed that QS6 leaves contained the greatest number of phenolic compounds comparing with QS2 and QO7 (Figure 1B–D). In line with the previous study, ferulic acid, apigenin, luteolin, caffeic acid, and two quercetin derivatives quercetin 3-galactoside and quercetin 3-O-glucuronide, which are known as major metabolites, were detected in QS2, QS6, and QO7 leaves as well (Figure 1E). Moreover, caffeic acid, apigenein, luteolin, and quercetin 3-galactoside were DAMs in comparable groups ‘QS2 vs. QS6’ and ‘QS6 vs. QO7’ (Figure 2B,C). These results indicated that the major compounds in different perilla varieties were almost consistent. Interestingly, we found several flavonoids, such as 4′,7-Di-O-methylnaringenin, taxifolin, galangin, diosmin, morin, rutin, and glycitein, were uniquely isolated in QS6 leaves. In addition, cryptochlorogenic acid, which is a kind of phenolic acid, was specifically detected in QS2 leaves (Figure 1E). This study is the first to report these above-mentioned phenolic compounds in perilla. These results suggest that different perilla varieties have different compositions of phenolic compounds, and some phenolic compounds may be unique to some perilla varieties. Since the metabolites reported in this study have clear pharmaceutical effects on antiphlogosis and antioxidant, antitumor, and other properties [3,29,30], QS6 might be a good variety of perilla for utilization in pharmaceutics. Based on these, our data provided new insights into composition of phenolic compounds in perilla and proved that targeted metabolome analysis is an efficient way to identify good perilla varieties with high potential for use in food and medicine.

### 3.2. Candidate Genes Involved in Phenolic Compound Biosynthesis in Perilla

In plants, phenolic compounds are thought to be the most widely distributed secondary metabolites [31]. To date, the biosynthesis of plant phenolics is almost clear, and there is no doubt that different phenolic compounds are generated via several pathways. The Shikimate pathway, phenylpropanoid pathway, and flavonoid branch pathway are three main pathways resulting in the formation of phenolic compounds [31,32]. Shikimic acid is the core of the shikimate pathway, and several aromatic amino acids, including L-phenylalanine, L-tyrosine, and L-tryptophan, and phenolic compounds originate from shikimic acid [33,34]. Subsequently, shikimic acid can generate several hydroxybenzoic acids, such as *p*-hydroxybenzoic acid, protocatechuic acid, and gallic acid [14]. At the beginning of the phenylpropanoid pathway, L-phenylalanine is catalyzed by the key phenolic metabolism enzyme PAL and results in the formation of trans-cinnamic acid, which is a bridge between the metabolism of aromatic amino acids and phenolic compounds [35,36]. In higher plants, the condensation of *p*-Coumaroyl-CoA and three molecules of malonyl-CoA leads to the formation of chalcone. Then, the reaction of biosynthesis of flavonoids is catalyzed by CHS [17,37]. So far, flavonoid-formation-related enzymes have been widely studied in plants [31,38,39]. In perilla, most of the enzyme encoding genes involved in flavonoid biosynthetic pathway have been isolated and identified [2]. However, factors that potentially participate in phenolic compounds are still unclear.

In this study, we performed the targeted metabolome analysis of phenolic compounds in three perilla varieties and found that several compounds belonging to productions of the shikimate pathway, phenylpropanoid pathway, and flavonoid branch pathway were differentially accumulated or unique in QS2 or QS6 leaves (Figure 1 and Figure 8). Based on the integrated transcriptome and metabolome analyses, we screened out 14 genes, which could be divided into modules of the shikimate pathway, phenylpropanoid pathway, and flavonoid pathway. These genes displayed high positive or negative correlation with 15 phenolic compounds (Figure 7). Protocatechuic acid is a type of phenolic acid, which has a wide range of pharmacological activities including antioxidant, anti-inflammatory, neuroprotective, antibacterial, antiviral, anticancer, and so on [40]. In this study, we found chalcone-flavanone isomerase 1 (C2S51_024895) and flavonol synthase (C2S51_007988) encoding genes were positively and negatively correlated with protocatechuic acid biosynthesis (Figure 7 and Figure 8). Although chalcone-flavanone isomerase 1 and flavonol synthase are key enzymes in the flavonoid branch pathway, they were still highly correlative with the phenylpropanoid pathway, indicating that the shikimate pathway and flavonoid pathway were not independent and there might be a feedback loop between these two pathways. In addition, we found that several key enzymes in the flavonoid pathway, for instance, one 4CL, two CHI, two F3H, one FLS, two DFR, and one ANS, were isolated. They all showed different expression profiles in QS2, QS6, and QO7 leaves (Figure 8). In perilla, some studies have reported enzymes in the flavonoid biosynthesis metabolic pathway [41,42]. However, these studies did not use the newest reference genome of perilla and this is inconvenient for gene cloning. Additionally, their results did not show the complete flavonoid biosynthesis metabolic pathway. Our results were based on the newest reference genome of perilla, and it was easy to clone the relative genes. Moreover, our results further covered the gaps seen in previous studies.

In addition, we found several peroxidase-encoding genes, including peroxidase 10, 31, 43, and 45, were highly correlative with luteolin, *m*-coumaric acid, diosmin, and cyanin chloride (Figure 7B), indicating that peroxidase displayed a close relationship with the biosynthesis of phenolic compounds. In plants, a series of secondary metabolites, especially phenols, may act as reductant substrates of class III peroxidases [43,44,45]. Peroxidases are thought as ubiquitous enzymes in plant cell walls and vacuoles, which are also the target compartments for the accumulation of most secondary metabolites [46,47,48]. Given these, it was highly possible that luteolin, *m*-coumaric acid, diosmin, and cyanin chloride were substrates of peroxidases.

## 4. Materials and Methods

### 4.1. Plant Materials, Growth Conditions, and Sample Collection

Perilla varieties QS2, QS6, and QO7 were planted in an experimental field in Shaoguan University (the research center for engineering and technology of aromatic plants, 24°46′ N 113°40′ E). All plants were given a unified and coincident treatment. Samples in the experiments were collected from fresh leaves at the maturity stage of branches at the same height, and each sample set three biological replicates. The leaves of each variety were divided into two parts, one for targeted metabolic analysis and the other for the transcriptome sequencing. The leaves for targeted metabolic analysis were frozen in liquid nitrogen and ground to a powder, then stored at −80 °C. Samples selected for transcriptome sequencing were cleaned after harvesting and immediately frozen using liquid nitrogen and stored at −80 °C.

### 4.2. RNA Isolation and Library Preparation

Total RNA was extracted from perilla leaves using the TRIzol reagent (Invitrogen, Waltham, CA, USA) according to the manufacturer’s protocol. RNA purity and quantification were evaluated using the NanoDrop 2000 spectrophotometer (Thermo Scientific, Waltham, CA, USA). RNA integrity was assessed using the Agilent 2100 Bioanalyzer (Agilent Technologies, Santa Clara, CA, USA). cDNA libraries were constructed using VAHTS Universal V6 RNA-seq Library Prep Kit according to the manufacturer’s instructions.

### 4.3. RNA Sequencing and Differentially Expressed Genes Analysis

The libraries were sequenced on an Ilumina Novaseq 6000 platform (Industrial Blvd., Hayward, CA, USA), and 150 bp paired-end reads were generated. Raw reads of fastq format were firstly processed using fastp [49], and the low-quality reads were removed to obtain the clean reads. The clean reads were mapped to the reference genome of perilla (https://www.ncbi.nlm.nih.gov/genome/?term=PRJNA431002 (accessed on 18 March 2025)) using HISAT2 [50]. FPKM [51] of each gene was calculated and the read counts of each gene were obtained by HTSeq-count. Differential expression analysis was performed using the DESeq2 [52]. Q-value < 0.05 and Fold change > 2 or Fold change < 0.5 were set as the thresholds for significantly differentially expression genes (DEGs). Hierarchical cluster analysis of DEGs was performed using R (v 3.2.0) to demonstrate the expression pattern of genes in different groups and samples. Based on the hypergeometric distribution, GO and KEGG pathway enrichment analyses of DEGs were performed to screen the significant enriched term using R (v 3.2.0). Pearson’s correlations of samples were performed on variance-stabilized transformed values, while principal component analysis (PCA) was performed on regularized log values of read counts.

### 4.4. Quantitative Real-Time PCR Assay

RNA was extracted using the RNAprep Pure Plant Plus kit DP441 (TIANGEN BIOTECH (BEIJING) CO., LTD, Beijing, China), and the extracted RNA was reverse-transcribed using FastKing gDNA Dispelling RT SuperMix kit KR118 (TIANGEN BIOTECH (BEIJING) CO., LTD) after being qualified by NanoDrop detection. The primers were designed using primer 5.0 software, and perilla actin gene was employed as an internal control (Table S1). qRT–PCR was performed using 2 × SYBR Green qPCR Master Mix reagent (1708891, Bio–Rad, Hercules, CA, USA) using an iCyeler iQTM/Clooo system (Bio–Rad, USA). The qRT-PCR cycling conditions were as follows: predenaturation at 95 °C for 30 s, followed by 40 cycles of denaturation at 95 °C for 5 s and annealing/extension at 60 °C for 30 s. The final step included a melting curve analysis at 95 °C for 5 s, followed by 60 °C for 1 min. Each gene was amplified in triplicate. The relative expression levels of each gene were computed using the 2^−ΔΔCt^ method.

### 4.5. Targeted Metabolome Analysis

In this study, the targeted metabolome analysis of phenolic compounds in perilla leaves was performed. The sampling method was the same as the transcriptome analysis experiment, and five biological replicates were set up. For the methods of targeted metabolome analysis, 600 μL of ice-cold MeOH-water (2:1, *v*/*v*, containing interior standard) was added into the freeze-dried perilla leaves. Then, the mixture was crushed using a mixer mill (Wonbio-E, WONBIO BIOTECHNOLOGY, Shanghai, China) with a zirconia bead for 2 min at 60 Hz. Afterward, the whole samples were ultrasonically extracted for 20 min in ice-water bath, and then centrifuged at 4 °C (13,000 rpm) for 10 min prior to decanting of 200 μL of supernatant to sample vials. Next, 400 μL ice-cold MeOH-water (2:1, *v*/*v*, containing interior standard) was added into the residual sample. The samples were ultrasonically extracted for 20 min in an ice-water bath, and then centrifuged at 4 °C (13,000 rpm) for 10 min prior to decanting 200 μL of supernatant into sample vials. The two supernatants were combined and mixed well. Then, the mixed supernatant (400 μL) was dried under a nitrogen stream and re-dissolved in 200 μL of MeOH-water (7:18, *v*/*v*, containing interior standard), extracted by ultrasonic for 5 min in ice-water bath, and then filtered through a 0.22 μm organic phase pinhole filter for subsequent UPLC-MS/MS (AB Sciex Qtrap 5500/Nexera UHPLC LC-30A) analysis. Quality control (QC) samples were prepared by mixing all samples to determine the reproducibility of the obtained results. Liquid chromatography was performed on a Nexera UHPLC LC-30A (SHIMADZU, Kyoto, Japan). A Waters ACQUITY UPLC HSS T3 column (2.1 × 100 mm, 1.8 μm) was used for analysis. Injection volume was 5 μL. The mobile phase A was water, containing 0.1% formic acid, and the mobile phase B was ACN. Gradient conditions were as follows with 0.3 mL/min flow rate: 0–2 min, 0 B; 2–30 min, 0–50% B; 30–32 min, 50–95% B; 32–34 min, 95% B; 34–34.1 min, 0–100% B; 34.1–35.5 min, 0 B. All the samples were kept at 4 °C during the analysis, and the column temperature was set at 40 °C.

Mass spectrometry was performed on the AB SCIEX Selex ION Triple Quad™ 5500 System, with an electrospray ionization (ESI) source, operating in both positive and negative ion mode. Nitrogen was employed as the collision gas. Additional instrumental parameters were as follows: positive ion mode: CUR: 35Psi; EP: 10 V; IS: 5500 V; CXP: 10 V; TEM: 500 °C; Gas1: 60 Psi; Gas2: 50 Psi; negative ion mode: CUR: 35 Psi; EP: −10 V; IS: −4500 V; CXP: −20 V; TEM: 500 °C; Gas1: 60 Psi; Gas2: 50 Psi. Targeted metabolites were analyzed in schedule multiple reaction monitoring (SRM) mode. The MRM pairs, decluttering potentials (DPs), and collision energies (CEs) were optimized for each analyte. Data acquisitions and further analysis were conducted using Analyst software (https://sciex.com/products/software/analyst-software (accessed on 18 March 2025)). SCIEX OS-MQ software (https://sciex.com/products/software/sciex-os-software (accessed on 18 March 2025)) was used to quantify all metabolites.

Differentially accumulated metabolites (DAMs) were isolated based on the variable importance of the projection (VIP) ≥ 1 and |log2 (fold change)| ≥ 1 (*p*-value < 0.05). The functions of DAMs were further annotated using the KEGG compound database to determine the metabolic pathways.

## 5. Conclusions

This research provided new information on the types and amounts of phenolic compounds in different perilla leaves. The results extended our understanding of the biosynthesis of phenolic compounds and isolated 14 candidate genes that highly correlated with 9 DAMs in QS2, QS6, and QO7 leaves and 9 phenolic compounds only in QS6 leaves. In addition, we focused on key enzymes in shikimate pathway, phenylpropanoid pathway, and flavonoid branch pathway and screened out 16 enzyme encoding genes, which further enhanced the possibilities of gene cloning from perilla. In conclusion, our results provide valuable information on phenolic compound biosynthesis and the genetic resources for molecular breeding in *Perilla frutescens*.

## Data Availability

The transcriptome data were released in National Genomics Data Center (https://ngdc.cncb.ac.cn/?lang=en (accessed on 13 October 2024)) with the accession number CRA020281 and the targeted metabolome data were released in National Genomics Data Center with the accession number OMIX007760.

## Supplementary Materials

The supporting information can be downloaded at: https://www.mdpi.com/article/10.3390/ijms26072841/s1.

## Abbreviations

The following abbreviations are used in this manuscript:

4CL	4-coumarate-CoAligase
ANR	anthocyanidin reductase
ANS	anthocyanidin synthase
C4H	cinnamate 4- hydroxylase
cDNA	complementary DNA
CHI	chalcone isomerase
CHS	chalcone synthase
CYP73A	trans-cinnamate 4-monooxygenase
CYP75B1	flavonoid 3′- monooxygenase
CYP93C	cytochrome P450 93C
DAHPS	3-Deoxy-D-arabino-heptulosonate 7-phosphate synthase
DAM	differentially accumulated metabolite
DEGs	differentially expressed genes
DFR	dihydrofavonol-4-reductase
DHQD	3-Dehydroquinate dehydratase
DHQS	3-Dehydroquinate synthase
F3H	naringenin 3-dioxygenase
F6H	feruloyl-CoA 6-hydroxylase
FG2	flavonol-3-O-glucoside L-rhamnosyltransferase
FLS	flavonol synthase
FNS I	flavone synthase I
FPKM	fragments per kilobase per transcript per million mapped reads
GO	gene ontology
KEGG	Kyoto encyclopedia of genes and genomes
PAL	phenylalanine ammonia-lyase
PCA	principal component analysis
QC	quality control
RT-qPCR	Real-time quantitative polymerase chain reaction
RNA-Seq	RNA sequencing
UAGT	Anthocyanidin 3-O-glucosyltransferase
UDPG	UDP-glucosyltransferase
UGT	UDP-glycosyltransferases
UPLC-MS/MS	ultra-performance liquid chromatography/tandem mass spectrometry
VIP	variable importance of the projection
